# Anti-*Malassezia* Drug Candidates Based on Virulence Factors of Malassezia-Associated Diseases

**DOI:** 10.3390/microorganisms11102599

**Published:** 2023-10-20

**Authors:** Muriel Billamboz, Samir Jawhara

**Affiliations:** 1 INSERM, CHU Lille, Institut Pasteur Lille, U1167—RID-AGE—Facteurs de Risque et Déterminants Moléculaires des Maladies Liées au Vieillissement, University of Lille, F-59000 Lille, France; muriel.billamboz@junia.com; 2 JUNIA, Health and Environment, Laboratory of Sustainable Chemistry and Health, F-59000 Lille, France; 3 CNRS, UMR 8576—UGSF—Unité de Glycobiologie Structurale et Fonctionnelle, INSERM U1285, University of Lille, 1 Place Verdun, F-59000 Lille, France; 4 Medicine Faculty, University of Lille, F-59000 Lille, France; 5 CHU Lille, Service de Parasitologie Mycologie, Pôle de Biologie Pathologie Génétique, F-59000 Lille, France

**Keywords:** *Malassezia* species, virulence factors, drugs, natural compounds, antifungals

## Abstract

*Malassezia* is a lipophilic unicellular fungus that is able, under specific conditions, to cause severe cutaneous and systemic diseases in predisposed subjects. This review is divided into two complementary parts. The first one discusses how virulence factors contribute to *Malassezia* pathogenesis that triggers skin diseases. These virulence factors include *Malassezia* cell wall resistance, lipases, phospholipases, acid sphingomyelinases, melanin, reactive oxygen species (ROS), indoles, hyphae formation, hydrophobicity, and biofilm formation. The second section describes active compounds directed specifically against identified virulence factors. Among the strategies for controlling *Malassezia* spread, this review discusses the development of aryl hydrocarbon receptor (AhR) antagonists, inhibition of secreted lipase, and fighting biofilms. Overall, this review offers an updated compilation of *Malassezia* species, including their virulence factors, potential therapeutic targets, and strategies for controlling their spread. It also provides an update on the most active compounds used to control *Malassezia* species.

## 1. Introduction

*Malassezia* is a lipophilic unicellular fungus [1]. Many species of *Malassezia* can be found in the healthy skin microbiota of humans [2]. Under specific conditions, they are capable of causing severe cutaneous and systemic diseases in predisposed subjects [3]. *Malassezia* species can become pathogenic when the physical, chemical, or immunological properties of the skin are altered [3]. Many environmental and endogenous factors have been found to facilitate the transition of *Malassezia* yeasts from a commensal state to a pathogenic one such as high temperatures and humidity, greasy skin, sweating, heredity, and immunosuppressive conditions [4]. *Malassezia* species have been associated with a number of diseases affecting human skin, such as seborrheic dermatitis, pityriasis versicolor, *Malassezia folliculitis*, and catheter-associated sepsis [5].

Seborrheic dermatitis is caused by a non-specific immune response to *Malassezia* species, which trigger an inflammation in seborrheic areas such as the scalp, face, chest, back, axilla, and groin [6,7]. For instance, *M. globosa* and *M. restricta* strains that are commonly isolated from human skin have distinct profiles of proinflammatory cytokines production from epidermal cells. However, dandruff is a less severe form of seborrheic dermatitis, which is restricted to the scalp, and causes itchy, flaking skin without visible inflammation [8]. A pityriasis versicolor infection is characterized by the proliferation of *Malassezia* yeasts and the activation of hyphae formation, which lead to the superficial invasion of the stratum corneum [6]. *Malassezia folliculitis* is caused by *Malassezia* yeasts invading the pilo-sebaceous unit, leading to a dilation of the follicles [9]. In addition, inflammatory infiltrates, and clinical inflammation are caused by ruptures of the follicular walls [9]. Currently, ten *Malassezia* species have been isolated from human skin (Table 1). Culture and molecular analysis as well as mycobiome approaches led to the identification of *M. restricta* and *M. globosa* as the most predominant fungal species on human skin [10,11,12].

This review discusses how virulence factors contribute to *Malassezia* pathogenesis that triggers skin diseases. These virulence factors include *Malassezia* cell wall resistance, lipases, phospholipases, acid sphingomyelinases, melanin, reactive oxygen species (ROS), indoles, hyphae formation, hydrophobicity, and biofilm formation.

## 2. *Malassezia* Virulence Factors

### 2.1. Malassezia Cell Wall Resistance

In comparison to other yeasts, *Malassezia*’s cell wall is very thick in comparison with other yeasts, representing up 26 to 37% of the cell volume [21,22]. Most cell wall components consist of glycans (70%), protein (10%), and lipid (between 15 and 20%), with a small amount of nitrogen and sulfur [22,23]. Mittag et al. showed that an outer lamellar layer surrounds the cell wall [24]. This lamellar layer is involved in adhesion of *Malassezia* to the catheter and the human skin [24]. All *Malassezia* species with the exception of *Malassezia pachydermatis* are all lipid-dependent [25]. Thus, *Malassezia* species require exogenous lipids to grow, making lipids a critical component of their growth [24]. The high lipid content of *Malassezia* cell wall contributes to their mechanical stability, pathogenicity, resistance, and osmoresistance [26,27]. Consequently, *Malassezia* pathogens are protected from phagocytosis and can escape the immune response [26,27]. Additionally, fungal adhesion to host surfaces is further enhanced by the hydrophobicity of the lipid-rich cell wall [24].

### 2.2. Lipases, Phospholipases and Acid Sphingomyelinases

*Malassezia* species lack the genes encoding fatty acid synthase suggesting that they cannot synthesize de novo fatty acids [28]. As a compensatory survival mechanism, *Malassezia* species display a panel of genes that encode secreted hydrolases like lipases, phospholipases, and acid sphingomyelinases, which promote these yeasts to obtain fatty acids from outside their fungal cells [28]. 

Lipases are considered to be a major virulence factor for *Malassezia* pathogens due to their lipid predominance in the cell walls. The lipases catalyze the hydrolysis of triacylglycerols to yield free fatty acids, diacylglycerol, monoacylglycerol, and glycerol [29]. There has been evidence that *M. furfur* releases fatty acids from a variety of lipids [30,31]. *Malassezia* contains many genes encoding lipases, which indicates that the fungus is required to degrade host sebum triglycerides into fatty acids. These neoformed fatty acids not only support the growth of the yeasts, but also cause skin diseases [31,32]. *Malassezia* phospholipases hydrolyze glycerol-phospholipid ester bonds. Like *Malassezia* lipases, it has been shown that *Malassezia* produces a variety of extracellular phospholipases that are considered virulence factors [30,33]. In addition to lipases and phospholipases, Malassezia yeast contains acid sphingomyelinase that hydrolyzes membrane lipid sphingomyelin into ceramide and phosphorylcholine. It contributes to Malassezia growth and virulence [28].

### 2.3. Melanin, ROS and Indoles

The pigment melanin has been implicated in the virulence of several important fungal pathogens indicating that melanin promotes their survival in hosts and facilitates fungal infection [34]. Different studies showed that *Malassezia* yeasts produce melanin [35,36]. Additionally, the yeasts and hyphae of *Malassezia* in both hypo- and hyperpigmented pityriasis versicolor lesions are strongly reactive with melanin-specific antibody, supporting that both forms of pityriasis versicolor undergo in vivo melanization [35].

One of the virulence factors of *Malassezia* species relies on their ability to generate ROS [37]. Both *M. furfur* and *M. pachydermatis* exhibited reduced ROS production after terbinafine treatment evidencing that terbinafine antifungal agent acts as an oxygen species scavenger [37].

*Malassezia* yeasts produce indole alkaloids from tryptophan [38]. When tryptophan is the only nitrogen source, this pathway is most active [38]. Indoles derived from *M. furfur*, such as malassesin, indirubin, and indolo[3,2-b]carbazole (ICZ), act as potent ligands for the host aryl hydrocarbon receptor (AhR). Thus, the interaction between these indoles and AhR affects all epidermal cells that express this receptor [39,40,41,42]. In addition, tryptophan metabolites can cause melanocyte apoptosis or prevent respiratory bursts in neutrophils [43]. By engaging AhR pathway, *Malassezia* yeasts modulate the inflammatory response and lead to skin pathologies [44].

### 2.4. Hyphae Formation

The hyphal formation plays an important role in the pathogenesis of *Malassezia* species [45]. The scales of pityriasis versicolor lesions provide an ideal environment for hyphal growth, and these lesions often contain large numbers of hyphae [46]. Some environmental factors play a role in *Malassezia* filamentation. A high humidity level increases skin secretions, which, in turn, promotes the growth and filamentation of *M. furfur* that facilitates skin infection [45]. Youngchim et al. showed that *M. furfur* filamentation was induced most strongly at pH 4.6 supporting that an acidic skin pH promotes the attachment of human microbiota to the skin [45]. In addition, a higher level of sebum may induce the formation of *Malassezia* hyphae in individuals with hyperactive sebaceous glands [25].

### 2.5. Hydrophobicity and Biofilm Formation

Fungal cell wall architecture has been associated with cell surface hydrophobicity (CHS) status [47,48]. Hydrophobic cells have short and aggregated fibril structures of cell wall mannoproteins [48]. In contrast, hydrophilic cells have longer, evenly spaced, and radiating structures [47]. CSH enhances microbial adhesion enabling pathogens to produce a biofilm which is a microbial community protected by an extracellular polysaccharide matrix. Angiolella et al. showed that many *Malassezia* isolates possess hydrophobic properties, adhere to abiotic surfaces, and produce biofilms [49]. *Malassezia* is prone to biofilm formation, which is a serious health threat and may provide drug resistance and increase virulence. Pedrosa et al. showed that *M. furfur* from skin isolates form biofilms on epidermal surfaces in the in vitro model, and the amount of biofilm increases over time [50].

**Figure 1:**
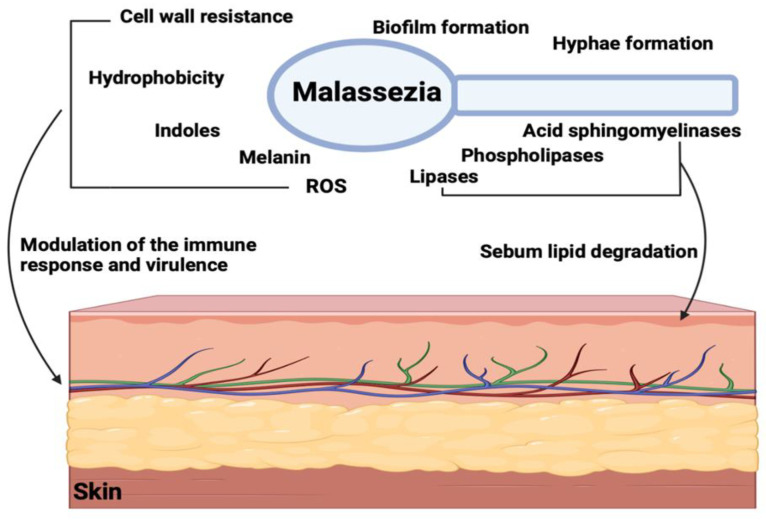
*Malassezia* virulence factors. Lipases, phospholipases and acid sphingomyelinases contribute to sebum lipid degradation into fatty acids which support *Malassezia* growth and cause skin diseases. Cell wall resistance, melanin, ROS, indoles, hyphae formation, hydrophobicity are involved in immune response modulation and contribute to *Malassezia* pathogenesis.

Figure 1 summarizes the main *Malassezia* virulence factors.

## 3. Antifungal Drugs against *Malassezia* Species

The prevalence of fungal infections worldwide has increased year after year due to the widespread use of immunosuppressive agents and the growing number of patients with critical infections [51]. Different studies evaluate the susceptibility of commercialized drugs to clinical *Malassezia* strains. *Malassezia* isolates are identified by morphological and biochemical criteria. The activity of reference drugs such as azoles (fluconazole, itraconazole, ketoconazole, voriconazole, luliconazole), or amphotericin B was assessed in vitro [52,53,54]. Rodrigues Silva et al. reported that *Malassezia* yeasts were susceptible to all tested drugs. However, around 1 in 10 strains exhibited high MIC for fluconazole and 1 in 100 strains showed resistance to several drugs [55]. Applying the same experiment design, Cafarchia et al. proved that fluconazole-resistant *M. pachydermatis* isolates exhibited cross-resistance to other azoles. This study underlined the importance of diversifying therapeutic options to fight fungal infections [56].

The purpose of this section is to describe active compounds that target identified virulence factors, such as thick membranes. Additionally, specific enzymes needed for yeast replication, like β-carbonic anhydrase or secreted lipases, are also interesting targets. Since *Malassezia* species are particularly lipophilic, this section will discuss how these drugs balance their hydrophilic/lipophilic properties.

**Scheme 1:**
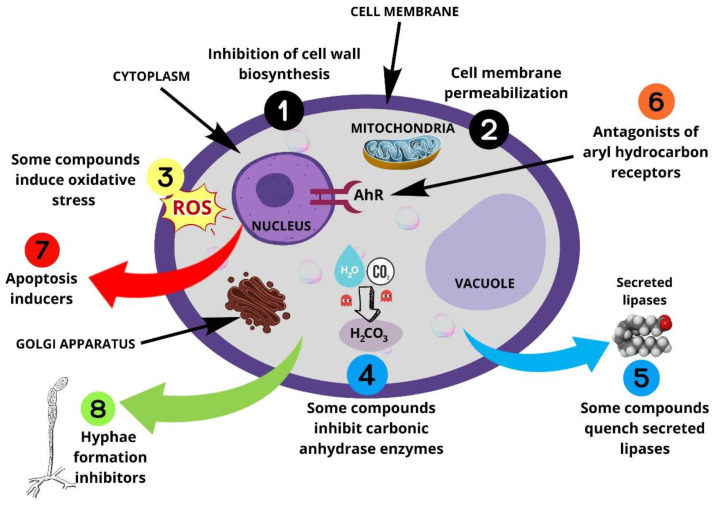
Strategies to tackle *Malassezia*-related infections.

Scheme 1 describes the different strategies used to tackle *Malassezia*-related infections.

### 3.1. Drugs Targeting Cell Wall and Membrane

Fungal cell walls are composed of glucans, chitin, mannans, glycoproteins and/or galactomannans, and chitosan. Their proportions and assembly depend on species. Therefore, drugs can impair the fungal cell wall and its biosynthesis. Table 2 summarizes the different biological pathways that can be disrupted along with some active compounds. 

#### 3.1.1. Inhibition of the Cell Wall and Membrane Biosynthesis

**Figure 2:**
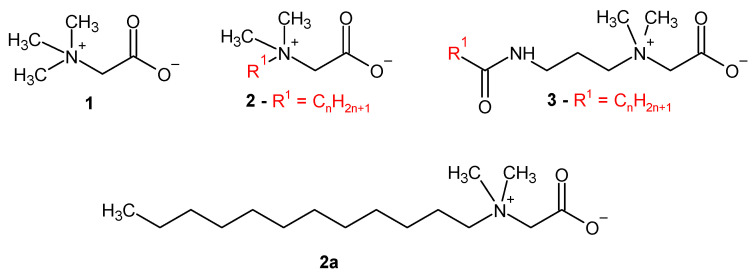
Chemical structures of betaine **1** and betaine derivatives **2** and **3** as described in [57].

Alkyl betaines are synthetic products obtained from betaine. Betaine **1**, also known as trimethylglycine, is a natural compound used in the human diet and comes from sugar beet (*Beta vulgaris*). Alkyl betaines **2,** or alkylamidopropylbetaines **3** are often introduced in shampoos as antistatic and viscosity-increasing agents (Figure 2). Hee Jung et al. tested the antifungal activity of alkyl betaines against *M. restricta* KCTC 27527 [57]. Among the six betaine derivatives, lauryl betaine **2a** exhibited high activity with MIC = 1.5–3 mg/mL. Using *Cryptococcus neoformans* as a phylogenetically close model, the authors showed that **2a** impaired cell membrane synthesis by affecting ergosterol production.

**Figure 3:**
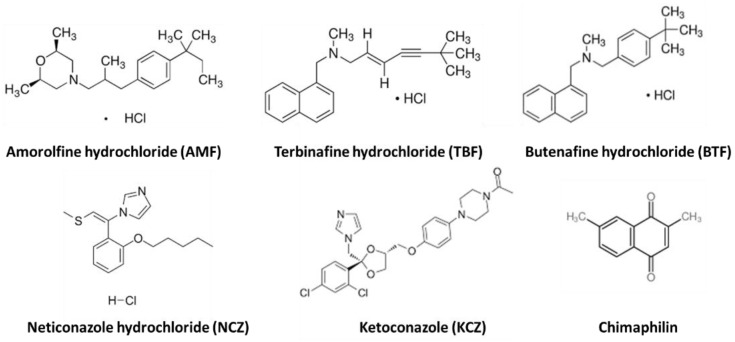
Chemical structures of drugs classified as ergosterol biosynthesis inhibitor used by Nimura et al. [58] and Chimaphilin [59].

**Figure 4:**
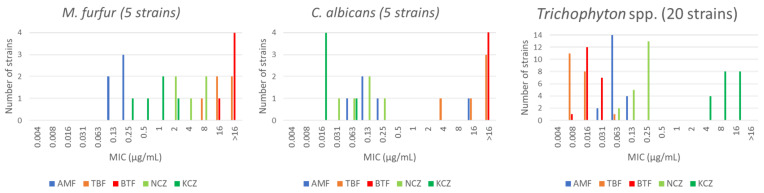
Biological results obtained used by Nimura et al. [58] and comparing the susceptibility of 5 drugs against a panel of strains.

Amorolfine hydrochloride (AMF), terbinafine hydrochloride (TBF), butenafine hydrochloride (BTF), neticonazole hydrochloride (NCZ), and ketoconazole (KCZ) are filed drugs classified as ergosterol biosynthesis inhibitors and have been used for three decades for dermatomycoses (Figure 3). In their comparative study, Nimura et al. tested them against their different strains, such as *Candida albicans*, *M. furfur,* and *Trichophyton* spp. Surprisingly, even if all these antifungal agents were supposed to have the same biological target, they clearly exhibited different antifungal potency. The two azole compounds (NCZ and KCZ) were active against *M. furfur*, whereas allylamine TBF and benzylamine BTF failed to inhibit *M. furfur*. The morpholine derivative AMF was the only drug that was fully active against all strains and could be called broad-spectrum antifungal [58]. The susceptibility of the fungal strains against this panel of drugs is represented in Figure 4. As shown, the antifungal properties of AMF, TBF, BTF, NCF, and KCZ strongly differed. This could be due to the difference in their chemical structures but also to the specific targets within the sterol biosynthesis. Three sub-groups can be defined according to their mode of action. TBF and BTF specifically inhibit the activity of the squalene epoxidase enzyme that is crucial in the formation of sterols necessary for fungal cell membranes (represented in red and orange, respectively, in Figure 4). Fungi treated with those drugs accumulate squalene while becoming deficient in ergosterol. As squalene epoxidase is not an enzyme of the cytochrome P-450 type, there is no potential inhibition of this class of enzymes. The morpholine derivated drug AMF (blue in Figure 4) mode of action is different and relies on the inhibition of two fungal enzymes: D14 reductase and D7-D8 isomerase, which are implicated in sterols biosynthesis. NCZ and KTZ (pale and dark green in Figure 4) block the synthesis of ergosterol, a key component of the fungal cell membrane, through the inhibition of cytochrome P-450 dependent enzyme lanosterol 14α-demethylase. By observation of the MIC against the panel of fungal strains, it is obvious that targeting the squalene epoxidase enzyme is more efficient in Tricophyton spp. Than in *C. albicans* or *M. furfur*. In contrast, KCZ and NCZ are potent antifungal agents against *C. albicans* and *M. furfur*. As far as *M. furfur* is concerned, AMF seemed the best antifungal agent. Its structure and mode of action could inspire the development of next-generation antifungal drugs. Chimaphilin, also known as 2,7-dimethyl-1,4-naphthoquinone, is the principal ingredient in *Chimaphila umbellata* (L.) W. Bart (Pyrolaceae) ethanol extracts (Figure 3). Chimaphilin exhibited antifungal activity against *M. globosa* (MIC = 0.39 mg/mL) and *M. restricta* (MIC = 0.55 mg/mL). Chemical-genetic profiling of chimaphilin indicates that this drug targets cell wall biogenesis and transcription pathways [59]. 

#### 3.1.2. Permeabilization of the Cell Membrane

##### Natural Compounds

**Figure 5:**
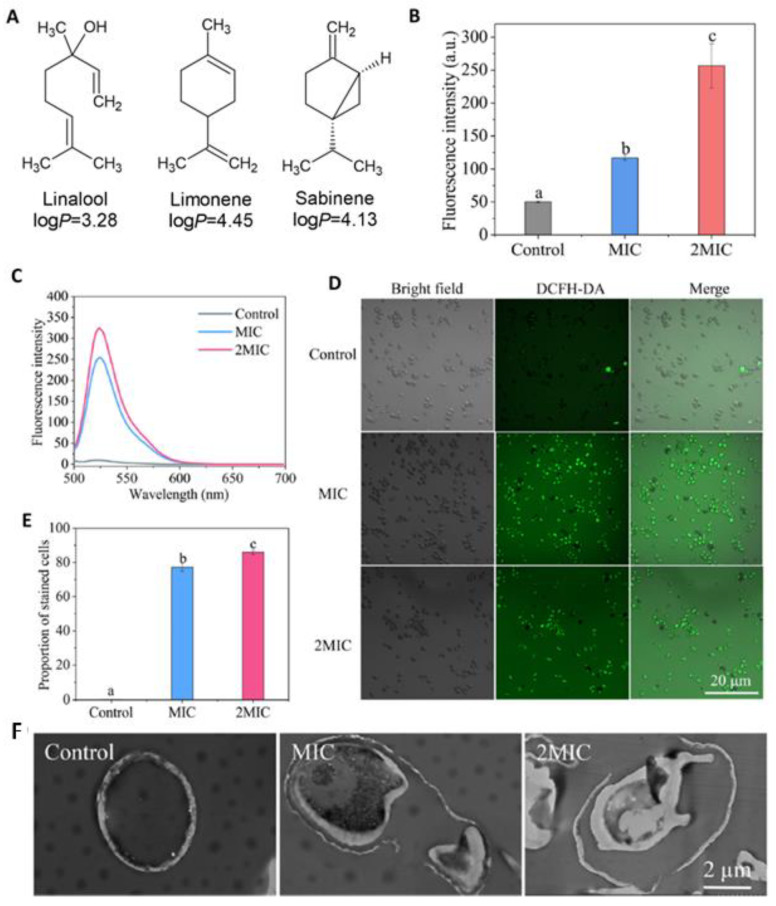
(**A**) Main components from *Zanthoxylum schinifolium* Siebold & Zucc. essential oil (ZSEO); (**B**) *M. restricta* was treated with different concentrations of ZSEO for 1 h and cell membrane fluidity changes were measured; (**C**) *M. restricta* was treated with different concentrations of ZSEO for 4 h and then intracellular reactive oxygen species (ROS) were measured with fluorescence spectrophotometer, confocal laser scanning microscopy (CLSM) (**D**), and flow cytometry (**E**); (**F**) *M. restricta* was treated with different concentrations of ZSEO for 4 h and then observed with transmission electron microscope (TEM).

*M. restricta* strains are becoming increasingly resistant to antifungal agents due to their resistance profile. *Zanthoxylum schinifolium* Siebold & Zucc. Essential oil (ZSEO) was investigated by Kan et al. [60]. Linalool (34.15%), limonene (21.24%), and sabinene (10.39%) were the main ingredients of ZSEO. ZSEO showed efficient antifungal activity against *M. restricta* with MIC and MFC values of 2.5 and 10 mg/mL, respectively. This result could be attributable to linalool, which is itself an antifungal agent (MIC = 2.5 mg/mL), more than to limonene or sabinene, which both displayed MIC values superior to 20 µg/mL. Low content components, such as α-pinene, β-ocimene, myrcene, terpinolene or hotrienol could also act as antifungals. To elucidate ZSEO’s mode of action, Kan et al. performed a series of experiments. Cell membrane damage assays revealed interactions between ZSEO and the cell membrane. This led to an increase in Zeta potential and depolarization of the cell membrane (Figure 5). ZSEO provoked a perturbation of membrane lipid organization, resulting in greater cell membrane fluidity (Figure 5). Moreover, the level of oxidative stress of *M. restricta* upon ZSEO treatment was measured by the fluorescent oxidative stress-sensitive probe DCFH-DA. The accumulation of intracellular ROS is characterized by the increasing green fluorescence intensity of the probe. This has been quantified by spectrophotometry, indicating a strong ROS accumulation (Figure 5). Overall, ZSEO interfered with cell metabolism, disturbed ROS homeostasis, and induced morphological and ultrastructural changes in *M. restricta* cells (Figure 5).

**Figure 6:**
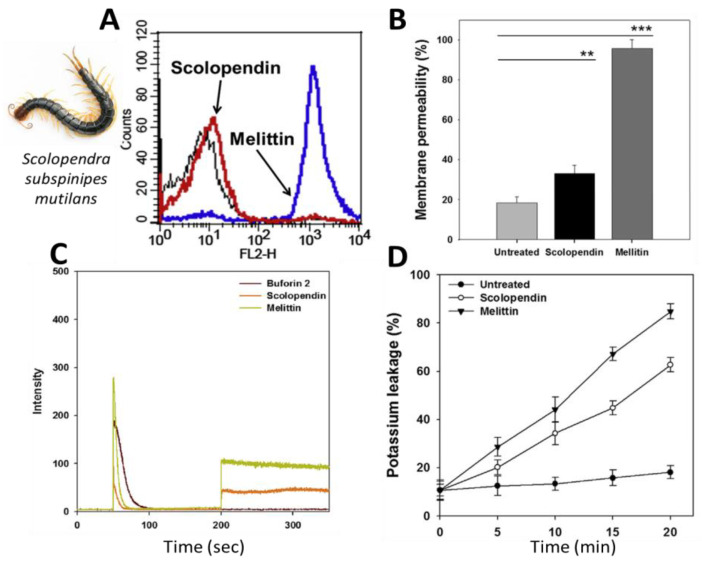
(**A**) *C. albicans* treated with scolopendin or melittin are stained with propidium iodide to detect increased membrane permeability. In black, the data from the blank. (**B**) The data represent the mean ± standard deviation for three independent experiments. **p < 0.05, *** *p* < 0.001 (Student’s *t*-test); (**C**) Depolarization of the membrane potential of *C. albicans* was detected by using DiSC3(5) dye. DiSC3(5) was added at t ¼ 50 s and the MIC of antimicrobial peptides at t ¼ 200 s to monitor fluorescence changes; (**D**) Potassium leakage after incubation with each AMP at its MIC in *C. albicans*. The error bars represent the standard deviation (SD).

Scolopendin, a novel cationic antimicrobial peptide (AMP), was isolated and characterized from the centipede *Scolopendra subspinipes mutilans* [61]. Lee et al. evaluated its antimicrobial activity against a panel of pathogenic microorganisms, including *M. furfur* KCTC 7744. Scolopendin exhibited a MIC value of 25 µM, which was lower than the one of Mellitin used as reference (MIC = 3.1–6.3 µM). This AMP did not produce hemolyze human erythrocytes even at 100 µM whereas Mellitin fully hydrolyzed them at the same concentration. Through the combination of several assays, the mode of action of scolopendin was elucidated on a *C. albicans* model strain and relied on its ability to interact with the microbial membrane and create pores (Figure 6).

The nuclear entry inhibitory signal peptide of HIV-1 Rev protein (Rev-NIS) showed potent antifungal activity against *M. furfur* (MIC = 20 µM) without hemolytic effects. Gathering a panel of assays, its mode of action was elucidated and relied on the depolarization and disruption of fungal membranes [62].

##### Synthetic Compounds

**Figure 7:**
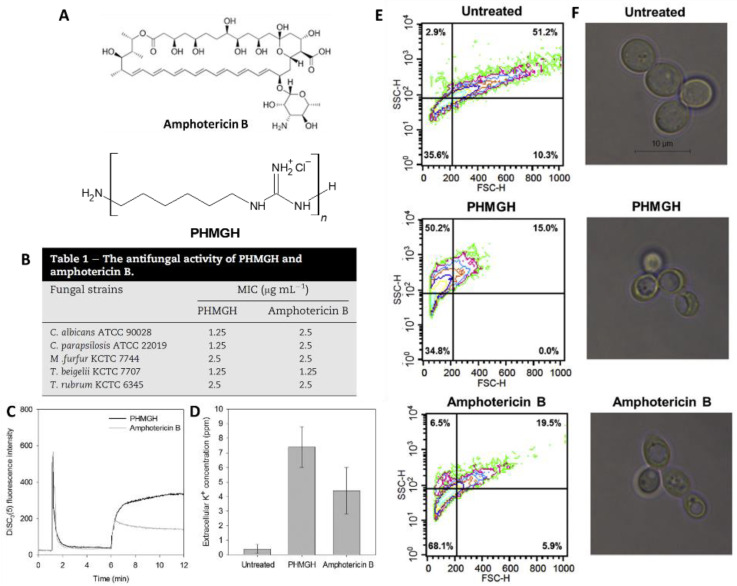
(**A**) The chemical structures of PHMGH and amphotericin B; (**B**) The antifungal activity of PHMGH and amphotericin B; (**C**) Membrane depolarization detected by a potential-sensitive dye, DiSC3 (excitation: 622 nm, emission: 670 nm). DiSC3 was added at t = 1 min. The compounds were added at t = 6 min; (**D**) KD leakage from yeast cells treated with 1.25 mg/mL compounds for 2 h at 28 °C. The data display the mean ± standard deviation for three independent experiments; (**E**) Flow cytometric contour-plot analysis of yeast cells treated with PHMGH or amphotericin B. SSC (y-axis, log value) represents cell granularity, and FSC (x-axis) represents cell size; (**F**) The morphological change was visualized by a microscope.

The cationic antimicrobial polymer-polyhexamethylene guanidine hydrochloride (PHMGH) has been described as a potent antifungal against *Malassezia furfur* KCTC 7744 with MIC = 2.5 µg/mL, being as active as amphotericin B [63]. This compound is positively charged and highly hydrophobic (Figure 7). After PHMGH treatment, the cells changed size and granularity. The membrane permeabilization was confirmed by ion transition assays. This showed that PHMGH exerted fungicidal activity by producing pores through the cell membrane. PHMGH did not show any hemolytic activity against human erythrocytes even at concentrations up to 16 × MIC. In contrast, amphotericin B destroyed erythrocytes at the same concentration. The mode of action of PHMGH is based on the cationic guanidinium groups that bind to the oxoanion phosphate group of the bacterial phospholipid membrane in a two-point hydrogen bonding chelate motif [64,65].

In the last two decades, less than a dozen studies investigated drugs that interfere with cell membrane biosynthesis or disrupt membranes. However, this thick and highly hydrophobic layer is responsible for the attachment of the cell to surfaces and subsequent proliferation. The yeast’s hydrophobicity has been identified as a major virulence factor. Studies on lipophilicity management in drug development have been conducted. The impact of maintaining a hydrophilic/lipophilic balance will be demonstrated in the following sub-part.

### 3.2. Impact of Hydrophobicity in Drug Ability towards Malassezia spp.

**Figure 8:**
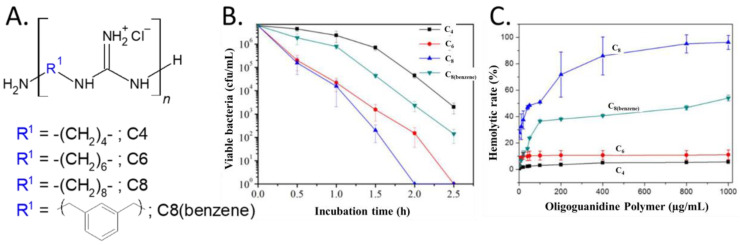
(**A**) Chemical structures of four oligoguanidine polymer analogs: Polybutamethylene guanidine hydrochloride (C4); Polyhexamethylene guanidine hydrochloride (C6); Polyoctanethylene guanidine hydrochloride (C8); Poly(m-xylylene guanidine hydrochloride) (C8(benzene)); (**B**) Time-kill curve of synthesized Polymer C4, C6, C8, C8(benzene) against a model strain. Approximately 6.25 × 10^6^ CFU/mL bacterial cells were incubated, respectively, with 32 μg/mL polymer at 25 °C for 0.5, 1, 1.5, 2, and 2.5 h; (**C**) Hemolytic properties of synthesized oligoguanidine Polymer C4, C6, C8, C8(benzene) against human erythrocytes adapted from [66].

It is crucial to optimize the hydrophilic/lipophilic balance (HLB) of a compound in order to optimize its intrinsic antifungal properties [66]. Zhou et al. demonstrated that an appropriate amphiphilicity balanced by the alkyl chain length, and the positive charge is an essential factor for the biocompatibility of cationic antimicrobial guanidine polymer. By studying four chemically related oligoguanidine polymer derivatives (Figure 8), the authors linked the alkyl carbon chain length with biocidal activity of the polymer and increased hemolytic activity, calcein dye leakage rate, and membrane interactions. Thus, intermediate compounds that disrupt the cytoplasmic phospholipid membrane of bacteria while having limited hemolytic effects on eucaryotic cells are preferred. Overall, polymers with optimal amphiphilicity, which are selected by varying the length of the alkyl chains, the positive charge, and their spatial arrangement, selectively target bacterial membranes instead of mammalian membranes [67,68].

The importance of lipophilicity has also been demonstrated by Supuran et al. for the design and evaluation of dithiocarbamate derivatives as β-class carbonic anhydrase inhibitors, as described in the title subpart [69].

Management of hydrophobicity helps to control drug delivery and efficacy. A number of recent studies have demonstrated that nanoparticles can increase antifungal activity against *Malassezia* species.

In 2020, Zhang, Lv et al. described the impacts of ketoconazole-lecithin-zein nanoparticles compared to free ketoconazole (KCZ) [70]. In the treatment of skin fungal diseases, KCZ is a widely used antifungal. However, it exhibits some limitations, such as low concentration, short efficacy duration, and severe systemic toxicity. Core-shell structures containing KCZ trapped in lecithin-zein nanoparticles (KLZ-NPs) allowed us to overcome these issues. Indeed, the in vitro penetration of KLZ-NPs in the stratum corneum and in deeper layers of skin increased. This improved skin distribution coupled with lower concentrations of KLZ-NPs in the spleen and liver, resulting in limited side-effects. 

KCZ encapsulation was also reported by Kaur et al. one year later [71]. KC loaded solid lipid nanoparticles (KTZ-SLNs) were prepared and evaluated. Spherical nanoparticles, displaying a mean size of 292 ± 6.3 nm and an optimal zeta potential equal to—24.39 exhibited a strong antifungal ability against *M. furfur*. KTZ-SLNs showed a 75–95% reduction in MIC value compared to the free-drug suspension (KTZ-SUS). Moreover, adding the lipidic shell allowed a 12.6-fold higher penetration up to the dermal layer of the skin. In addition, such nanoparticles showed high biocompatibility and hemocompatibility profiles. 

Following the same objective, carvacrol nano-based delivery systems were designed [72]. Altogether, nanoparticles increased hydrophobicity [73].

### 3.3. Impacting Nuclear Aryl Hydrocarbon Receptors by AhR Antagonists

The aryl hydrocarbon receptor (AhR) is a nuclear receptor and transcriptional regulator with multifaceted biological functions. *Malassezia* yeasts produce potent AhR ligands, such as indirubin (IND), indolo[3,2-b]carbazole (ICZ), tryptanthrin, and malassezin, has been linked with its pathogenesis. For example, malassezin induces apoptosis in primary human melanocytes [74]. Janosik, Begman et al. recently reviewed the chemistry and properties of indolocarbazoles, underlying their involvement in numerous biological pathways [75]. Therefore, proposed molecules able to block these activators may lower *Malassezia* spp. virulence.

**Figure 9:**
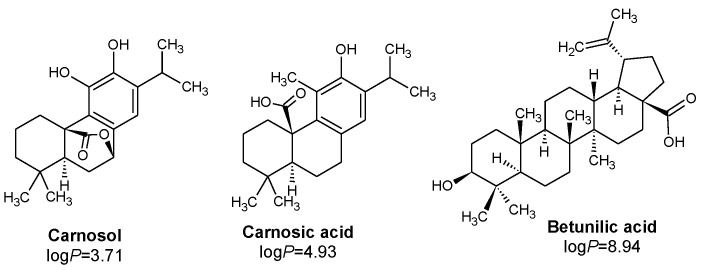
Chemical structures of effective antagonists of the AhR.

Only a few studies on AhR antagonists have been conducted in *Malassezia* species to date. In 2022, Magiatis et al. described the capacity of *Rosmarinus officinalis* L. Leaf Extracts (ROE) and their metabolites to inhibit AhR activation. The measurements were performed both in vitro (guinea pig cytosol) and in human keratinocytes. The prototype ligands 2,3,7,8-tetrachlorodibenzo-p-dioxin (TCDD) and Malassezia ligands were used to activate AhR. In such assays, metabolites of *R. officinalis* L., as abietane derivatives carnosol, carnosic acid, and triterpene betulinic acid, exhibited dose-dependent antagonist activity against TCDD (Figure 9) [76].

### 3.4. Targeting Enzymes Implied in Cell Survival

Few strategies and treatments combat *Malassezia* species, most of which target fungal growth. Drug resistance has led to alternative approaches. Several alternative approaches have been suggested, including inhibition of enzymes, such as the β-carbonic anhydrase (CA, EC 4.2.1.1) or the lipase secreted by *Malassezia* species.

#### 3.4.1. β-Class Carbonic Anhydrases

Carbonic anhydrases (CAs, EC 4.2.1.1) are metalloenzymes belonging to seven genetically distinct families. The active sites of these enzymes are usually zinc-containing, but ferrous iron, cadmium, and zinc may also be present, depending on the organism. Many of these enzymes can be inhibited pharmacologically [77,78,79]. Over the years, synthetic and natural compounds have been reported.

##### Synthetic Compounds

**Figure 10:**
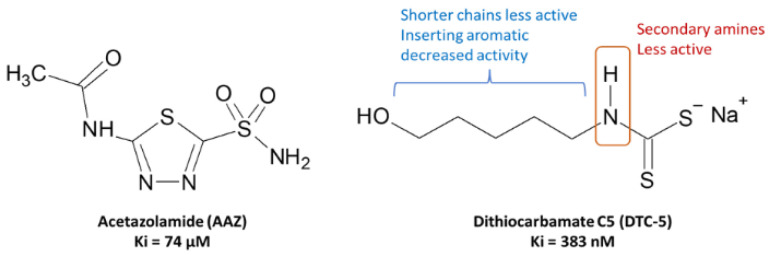
Structure of β-carbonic anhydrase inhibitors: acetazolamide AAZ and DTC derivative (DTC-5).

Using dithiocarbamate derivatives (DTCs) as an inhibitor, Supuran et al. demonstrated that it was able to inhibit a β-class carbonic anhydrase (CA, EC 4.2.1.1) from *M. globosa* (MgCA) [69]. DTCs are bioactive compounds largely used in varied medicinal applications, due to their ability to form chelates and interact with metalloenzymes active sites [80]. All DTCs proved to be more effective than the reference drug acetazolamide AAZ with Ki ranging from 383–6235 nm compared to Ki = 74 µM for clinically used AAZ (Figure 10). For aliphatic DTC derivatives, lipophilicity was correlated to antifungal activity (Figure 10).

Surupan and Nocentini groups are actively involved in the design and evaluation of new compounds as β-carbonic anhydrases inhibitors. Following their preliminary work on *N*-nitrosulfonamides [81], in 2019, they synthesized a series of benzenesulfonamides bearing nitrogenous bases, such as uracil and adenine, and evaluated their activity against *M. globosa* carbonic anhydrase (MgCA). These compounds displayed low Ki with values ranging from 138.8 nM to 5012.8 nM. The most active compound was the adenine derivative AD (Figure 10). According to this study, designed compounds inhibited strongerβ-carbonic anhydrases from *Candida glabrata* (CgNce103) and *Cryptococcus neoformans* (Can2) [82]. 

**Figure 11:**
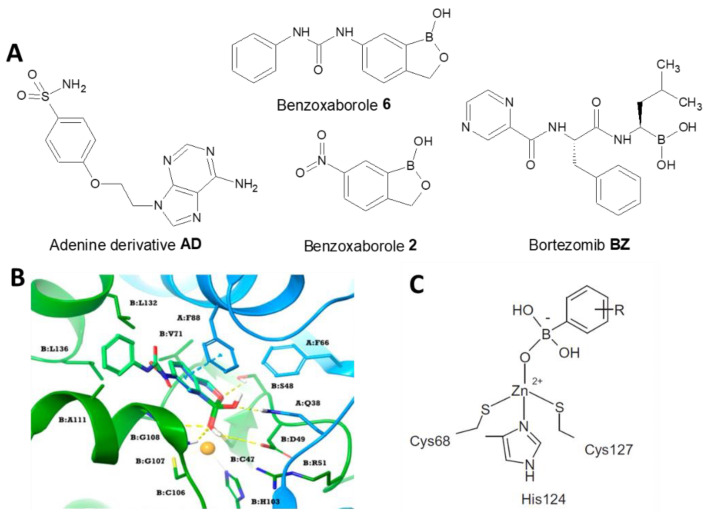
(**A**) Chemical structures of designed compounds adenine derivative from [82] and benzoxaboroles **2** and **6**; (**B**) Predicted binding modes of 2 (light blue) and 6 (green), within MgCA active sites. The nitro group of 2 established lipophilic contacts with B:A111, B:L132, and B:G107. The ureido group of 6 was involved in a three-center H-bond with B:G107.(Reprinted with permission from [84]. Copyright 2023 American Chemical Society); (**C**) Proposed binding of aromatic boronic acids to β-CAs. In β-CAs, the Zn(II) is coordinated by one His and two Cys residues (Can2 numbering system used in **C**), and the fourth ligand being a water molecule/hydroxide ion, which being a strong nucleophile may react with the electrophilic boronic acid leading to the adduct.

Boron molecules are increasingly studied in medicinal chemistry, as antifungals [83]. In 2017, the same team described benzoxaboroles’ activity. As for benzenesulfonamides, all reported derivatives showed nanomolar inhibitory activities against Can2 and CgNce103 vs. micromolar inhibition against MgCA (Figure 11). The selectivity for fungal CA towards human CA was fair to excellent. MgCA’s active site interactions were predicted using molecular modeling (Figure 11). Chemically, modification of the ureido by a thioureido group did not impact inhibitory activity. However, changes in the substitution pattern on the phenyl ring of the ureido/thioureido group result in differences in inhibitory activity and selectivity compared to hCA I and hCA II [84]. Bortezomid BZ, a boron derivative, was also investigated previously by Supuran as a CA inhibitor [85]. BZ inhibited MgCA at low micromolar concentrations. This compound interacts with CAs by chelating the zinc cation present in the active site (Figure 11).

**Figure 12:**
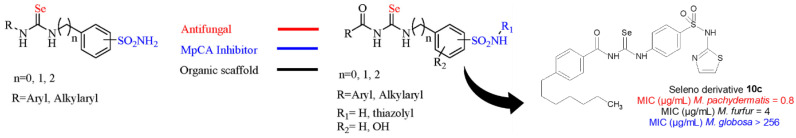
Design of seleno compounds with selective antifungal activity against *M. pachydermatis*, adapted from [86].

Recently, Surpuran et al. reported that seleno-containing molecules act as potent and selective antifungal agents against *M. pachydermatis*. Compared to oxygen or sulfur, the selenium atom increased antifungal activity. The sulfonamide moiety has been modified to be selective for *M. pachydermatis* compared to *M. furfur* and *M. globosa* from its related genus. A number of selenium-containing compounds displayed a safe toxicity profile and may be considered as promising antifungal agents (Figure 12) [86].

##### Natural Compounds

**Figure 13:**
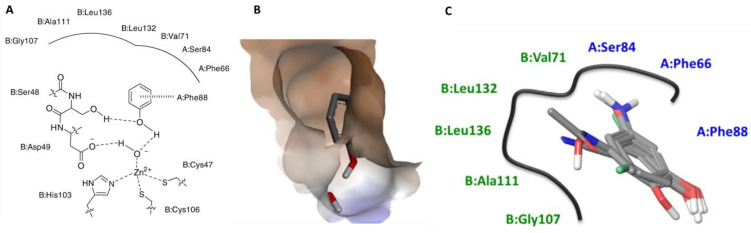
(**A**) Schematic representation of the binding mode of phenol into the MgCA active sites; (**B**) Hydrophobic surface of binding site receptor area; hydrophobicity increases from blue to brown; (**C**) Schematic representation of the aligned derivatives in the hydrophobic pocket.

In the same period, Supuran et al. proved that natural polyphenols, such as flavones, flavonols, flavanones, flavanols, or isoflavones exhibited MgCA inhibitory activity in the micromolar range. In addition, they showed high selectivity towards the fungal isozyme compared to its off-target human isoforms. Thanks to molecular docking investigations, they proposed that the natural polyphenols binding mode is based on zinc interaction through the hydroxyl group [87]. Synthetic phenols were also investigated. The hydroxyl group in the inhibitors anchored to the zinc-water coordinated in the active site and created hydrogen bonds with amino acid residues. Changing the phenyl group altered the hydrophobic pocket within MgCA’s active site [88] (Figure 13).

**Figure 14:**
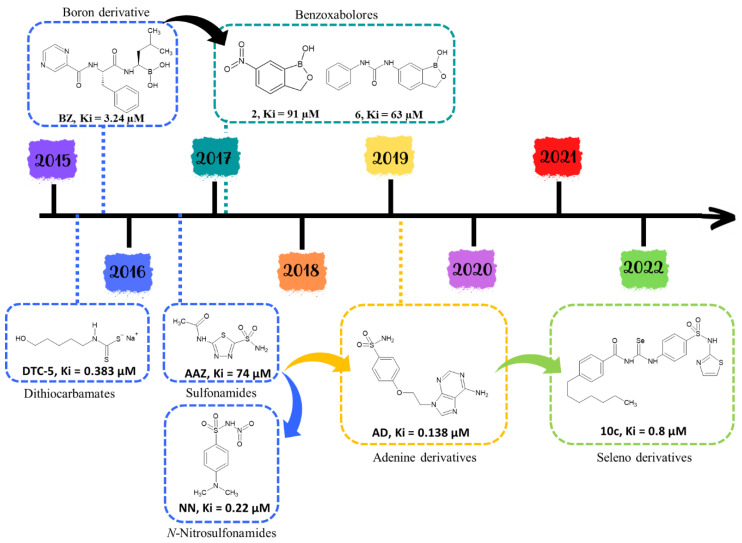
Timeline discovery of new chemical class as inhibitors of Malassezia β-carbonic anhydrases.

In summary, research conducted by th Supuran team has led to the design and evaluation of half a dozen chemical classes that can successfully inhibit Malassezia β-carbonic anhydrases. These derivatives are categorized into two main groups: anions, such as dithiocarbamates and hetero-compounds, bearing sulfur, boron, or selenium moiety. Sulfonamide derivatives are most abundant (Figure 14). A comprehensive overview of all compounds is available in [77]. Based on these reports, the design of novel entities is still ongoing and could help defeat resistant fungal infections due to *Malassezia* species.

#### 3.4.2. Quenching Secreted Lipases

Secreted lipase is an indispensable virulence factor to ensure fungal cell growth. The ability of *Malassezia* spp. to metabolize lipids and incorporate fatty acids into the cell wall is necessary for its pathogenicity. Therefore, quenching secreted lipases may be an effective method of stopping proliferation.

Karutha, Pandian et al. identified embelin as an antifungal that can inhibit lipase activity in *Malassezia* species [68]. A MIC of 400 µg/mL was observed for embelin, which resulted in a 65–89% reduction in lipase activity. Additionally, it showed synergistic effects when combined with ketoconazole [89]. 

**Figure 15:**
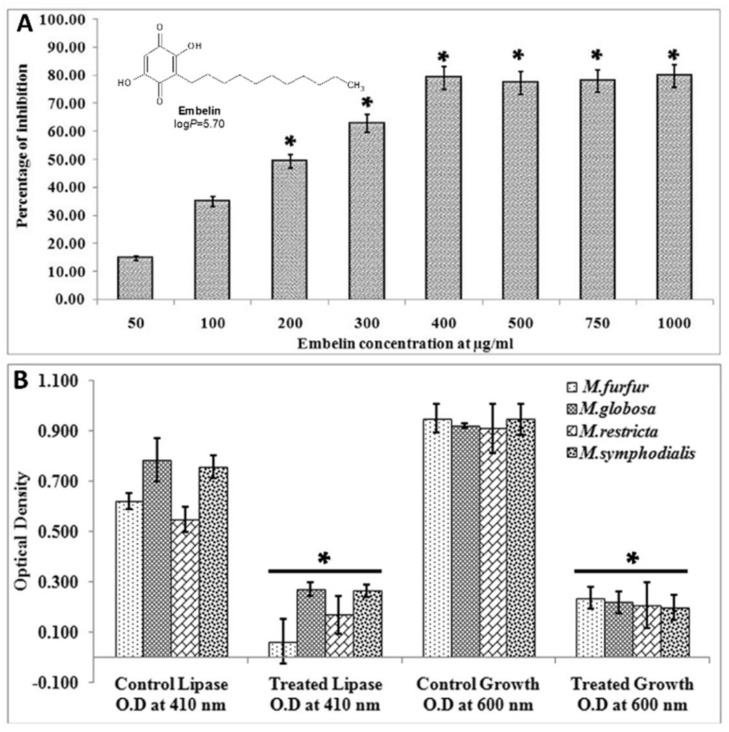
(**A**) Determination of minimal inhibitory concentration of embelin against *M. furfur* (MTCC 1374). Error bars and * symbol represent SD and statistical significance (* *p* < 0.05), respectively; (**B**) Effect of embelin on the secreted lipase of *Malassezia* spp.

The synergy between conventional drugs and natural products has already been described [90]. Additionally, embelin is highly lipophilic (logP = 5.70). Its long aliphatic chain (eleven carbons) may interact with the lipid membrane and disturb it. This long alkyl tail is like seleno-derivative 10c (Figure 15).

**Figure 16:**
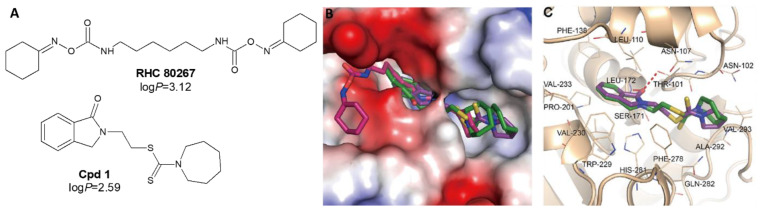
(**A**) Chemical structures of the compounds involved; (**B**) Docked binding poses of the compounds involved. (**B**) Superimposition of RHC 80267 with compounds **1**, **4**, and **5**, the lipase pocket is displayed as a charged surface. Docked binding poses of the compounds involved. (**A**) Superimposition of RHC 80267 with compounds **1**, **4**, and **5**, lipase pocket is shown as a charged surface. Binding poses of (**C**) compound **1** (green) and **4** (purple). Compounds are shown in sticks, interacting residues in SMG1 are depicted in lines, and hydrogen bonds are represented in dashed red lines.

One approach to relieving the *M. globosa* induced diseases relies on blocking the secreted lipase activity. *Malassezia globosa* LIP1 (SMG1), a representative secreted lipase from *M. globosa* CBS 7966. It represents a potential target. Wang et al. reported a docking-based virtual screening based SMG1 crystal structure and lipase assay [91]. These experiments were conducted to identify the first HIT molecule against SMG1 called Cpd 1 (Figure 16). RHC 80267, a well-known inhibitor of mammals’ lipases, was selected as a positive control. RHC 80267 exhibited an IC_50_ = 75.25 µM whereas Cpd 1 IC_50_ was 4-fold higher (IC_50_ = 20.09 µM). These results could serve as a starting point for the rational design of more potent inhibitors against SMG1.

Targeting secreted lipases to quell its activity is a novel approach to drug development against *Malassezia* spp.

### 3.5. Antioxidant Activities and ROS Attenuation

Pathogens from the *Malasseziae* genus cause persistent inflammation. To control diseases caused by *Malassezia* species, compounds that reduce ROS production and inflammation are of great interest. A number of routine assays can be performed, such as the DPPH assay and nitric oxide (NO) radical scavenging activity assay. A compound’s anti-inflammatory potential can also be determined by measuring the expression of interleukins or TLRs. Recently, only natural compounds, such as essential oils, fatty acids, and antimicrobial peptides, have been reported. 

#### 3.5.1. Extracts

**Figure 17:**
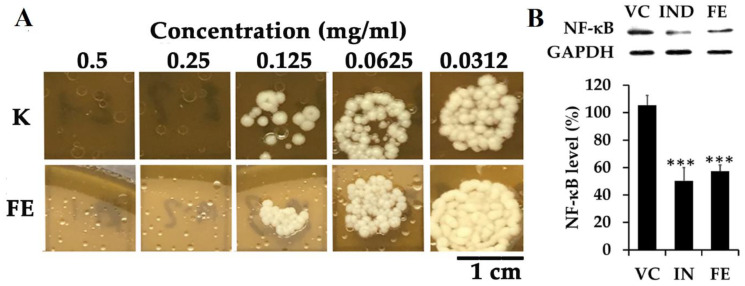
(**A**) *M. furfur* growth on modified Dixon agar after being treated with various concentrations ranging from 0.0312 to 0.5 mg/mL of ketoconazole (K) and fermented *O. sanctum* (FE) for 72 h at 32 ± 2 °C; (**B**) NF-kB levels after being treated with indomethacin (IND), bio-fermented *O. sanctum* extract (FE) in U937 cells for 48 h detected by Western blot analysis. A vehicle control (VC) was U937 cells treated with DMSO (1% *v*/*v*). *** *p* < 0.001.

In 2022, Chaiyana et al. reported the anti-inflammatory and antimicrobial activities of fermented *Ocimum sanctum* Linn. extract (FE) against a panel of microbial strains, including four different strains of *M. furfur* [92]. Nuclear factor kappa B (NF-kB) expression inhibition was measured using Western blot analysis. Table 3 and Figure 17A summarize the antifungal activities against *M. furfur*. Figure 17B shows the level of NF-kB after treatment of U937 cells. FE showed excellent activity, comparable to iodomethacin INC used as a positive control. Taken together, these data suggest that FE could be used as an alternative anti-inflammatory agent. In addition, it could be formulated as a topical antimicrobial agent against *M. furfur* to replace current chemical antimicrobial agents.

Kritzinger et al. reported the activity of nine plant ethanolic extracts against *M. furfur*. The 1,1-diphenyl-2-picrylhydrazyl (DPPH) and nitric oxide (NO) radical scavenging activities of these extracts were investigated. Among the tested panel, the root extract of *Rhoicissus tridentata* showed the highest antifungal activity against *M. furfur* with a 20.3 ± 4.7 mm zone of inhibition at a concentration of 2 mg/mL. *R. tridentata* extract displayed an IC_50_ = 2.3 ± 1.9 µg/mL DPPH radical scavenging activity, being as potent as ascorbic acid used as a reference. It also showed a fair NO radical scavenging ability with an IC_50_ = 306.2 ± 3.4 µg/mL, being 5-fold less active than vitamin C. Moreover, the absence of a cytotoxic effect on HaCat cells indicates that such extract could be explored for use as a topical treatment [93].

Chimaphilin (structure shown in Figure 3), also known as 2,7-dimethyl-1,4-naphthoquinone, is the principal ingredient in *Chimaphila umbellata* (L.) W. Bart (Pyrolaceae) ethanol extracts. Chimaphilin exhibited an antifungal activity against *Malassezia globosa* (MIC = 0.39 mg/mL) and *Malassezia restricta* (MIC = 0.55 mg/mL). *C. umbellata* crude extract showed strong antioxidant activity of measured using the DPPH (2,2-diphenyl-1-picrylhydrazyl) assay. The IC_50_ was determined at 142.1 ± 3.6 ppm, ten-fold less active than vitamin C (22.1 ± 0.1 ppm) or quercetin (17.2 ± 0.1 ppm). This assay suggests antioxidant ability of extract [59]. The diversity of cellular processes affected by chimaphilin suggests that the compound has multiple targets within the yeast cell. 

**Figure 18:**
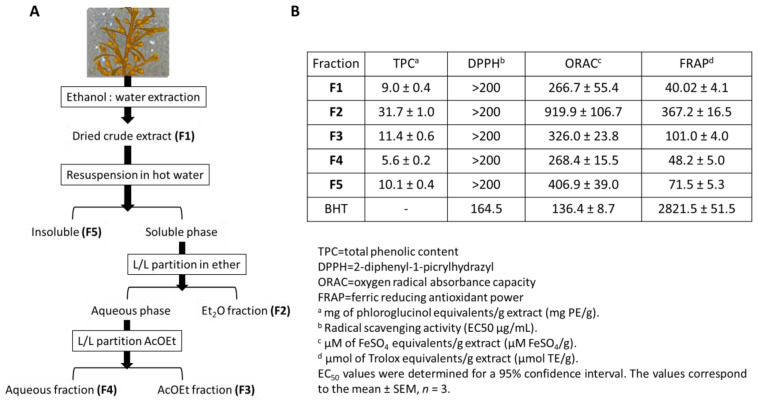
(**A**) *Sargassum muticum* extraction flowchart; (**B**) Antioxidant capacity of *Sargassum muticum* fractions adapted from [94].

According to Pedrosa et al. [94], seaweeds are a bioresource of active compounds. The antioxidant potential of *S. muticum* fractions was evaluated by the 2,2-diphenyl-1-picrylhydrazyl (DPPH) radical scavenging assay, the ferric reducing antioxidant power (FRAP), the oxygen radical absorbance capacity (ORAC) and its total phenolic content (TPC) was determined by the Folin–Ciocalteu method (Figure 18).

#### 3.5.2. Fatty Acids

**Figure 19:**
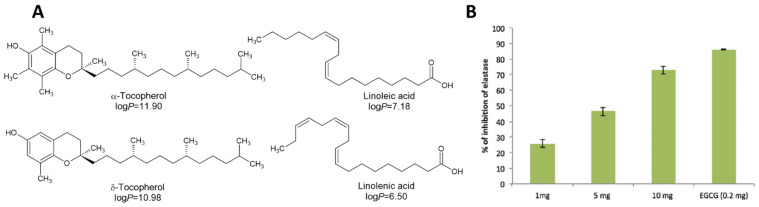
(**A**) Chemical structure and lipophily of main components of *Dittrichia viscosa* L. leaves lipid extract; (**B**) Elastase inhibition activity (%) of *D. viscosa* lipid extract at different concentration (1, 5, and 10 mg/mL) compared with EGCG.

The role of fatty acids in dietary supplementation is highly studied. Medium fatty acids have been widely reported for their metabolic features and cognition in humans [95,96,97] whereas long ones are fewer mentioned. Boulila et al. reported *Dittrichia viscosa* L. leaves lipid extract as an underestimated source of essential fatty acids and tocopherols with antifungal and anti-inflammatory activities [98]. The chemical composition of this lipid extract was determined by spectroscopic methods. Linoleic and linolenic acids were assessed as the main fatty acids while α- and δ-tocopherols were the most abundant tocopherols (Figure 19). All these compounds are highly lipophile with logP ranging from 6.5 to 11.9. This extract exhibited inhibition of a panel of *Malassezia* strains with ZOI ranging from 13 to 20 mm at 100 mg/mL even for fluconazole-resistant strains. This property of inhibiting elastase enzyme may be crucial to managing inflammation associated with yeast infections. This extract displayed anti-elastase activity (Figure 19B), increasing with concentration. Anti-elastase properties were similar to those of epigallocatechin gallate (EGCG) at a concentration of 10 mg/mL. The authors linked the richness of polyunsaturated fatty acids and tocopherols to this high anti-elastase capacity. However, the mechanism of elastase inhibition is still under-documented. These data must be related to those underlining the role of fatty acids and tocopherols in decreasing inflammation pathways [99,100,101] or the impact of additional fatty acids on *Malassezia* species’ growth [102]. In conclusion, this lipophilic extract may be effective in controlling inflammatory diseases and fungal infections.

#### 3.5.3. Antimicrobial Peptides (AMPs)

**Figure 20:**
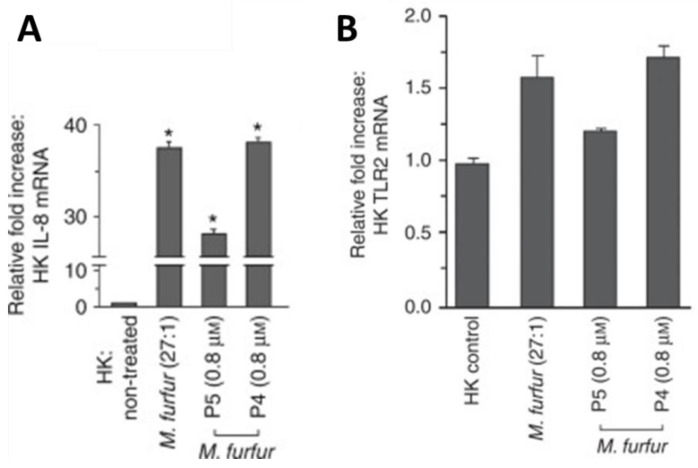
(**A**) Cecropin A–magainin 2 (CA–MA) hybrid peptide analog P5 inhibits the expression of IL-8 (**A**) and Toll-like receptor 2 (TLR2); (**B**) induced by *M*. *furfur* infection in normal human keratinocytes from [103]. * *p* < 0.001.

Because *M. furfur*-related diseases are problematic to treat and associated with increasing inflammation, the antifungal and anti-inflammatory effects of the antimicrobial peptide cecropin A(1–8)–magainin 2(1–12) hybrid peptide analog P5 on *M. furfur* were investigated. P5’ s minimal inhibitory concentration against *M. furfur* was 0.39 mM. This made it 3–4 times more potent than commonly used antifungal agents such as ketoconazole (1.5 mM) or itraconazole (1.14 mM). P5 does not induce eukaryotic cytotoxicity at its fungicidal concentration. P5 decreased the expression of Toll-like receptor 2 and IL-8 in *M. furfur*-infected human keratinocytes (Figure 20). In addition, P5 significantly downregulated NF-kB activation and intracellular calcium fluctuation, two pathways related to increased responses to keratinocyte inflammation induced by *M. furfur* infection [103].

### 3.6. Apoptosis Inducers

**Figure 21:**
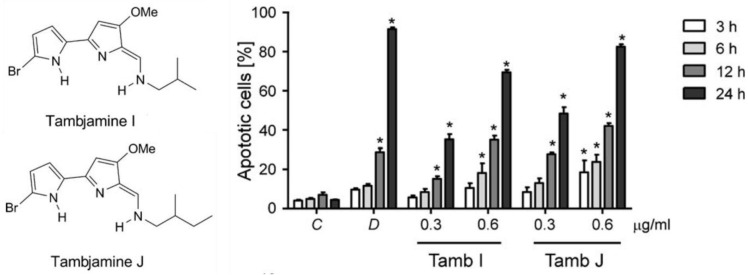
Apoptosis induced by Tambjamine I and J alkaloids as adapted from [113]. Negative control (C) was treated with the vehicle used for diluting the tested substance. Doxorubicin (D; 0.3 mg/mL) was used as positive control. * *p* < 0.001.

Apoptosis is a form of programmed cell death in multicellular organisms. Tambjamines are natural products belonging to the group of alkaloids. They are composed of a bipyrrole ring bearing an enamine moiety. The diversity of this nitrogen-substituent led to a large variety of derivatives and biological applications. From 2010, Tambjamine derivatives were reported with potent antimicrobial, anticancer, and antimalarial bioactivities [104,105,106,107,108,109,110,111,112]. In 2010, Banwell, Pessoa et al. reported the first anti-Malassezia potential of some synthetically derived tambjamines [113]. Most of them were more active than amphotericin B to eradicate *M. furfur* colonies. Tambjamine I and Tambjamine J displayed a zone of inhibition against *M. furfur* strains equal to 17 mm at a concentration of 0.1 mg/mL. Tambjamines I and J exhibited significant apoptosis-provoking effects (Figure 21), which is slightly similar to those of the Doxorubicin (D) used as a positive control. According to reported works, the biological effects of Tambjamines to induce apoptosis could be related to their anion transport ability [114,115,116,117,118] and their lipophilicity is considered as a key factor for activity [119]. 

Coprisin is a 43-mer defensin-like peptide from the dung beetle, *Copris tripartitus*. Coprisin exhibited high antifungal activity against *M. furfur* with MIC = 5 µM without any hemolytic side-effects. Like *C. albicans*, its mode of action is likely related to apoptosis. Indeed, coprisin did not induce rupture of the cell membrane but accumulates in the nucleus, inducing programmed cell death [120].

### 3.7. Targeting Hyphae Growth

**Figure 22:**
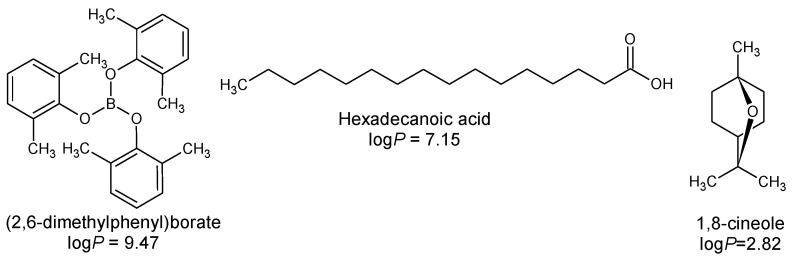
Chemical structures of main components from *A. galanta n*-hexane extract.

**Figure 23:**
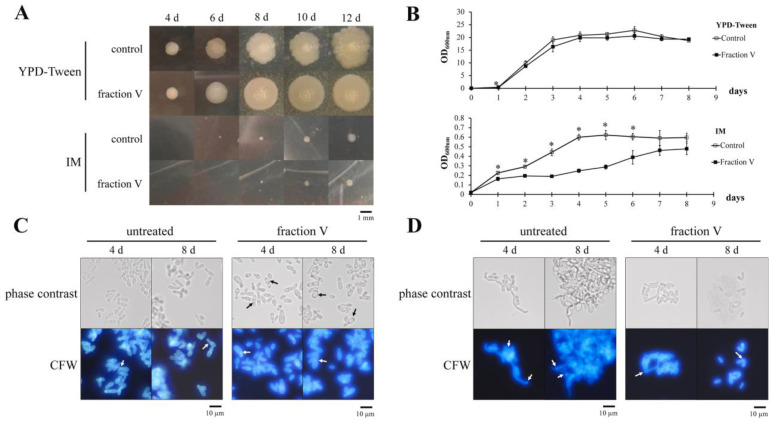
Effects of Fraction V on *M. furfur* at 1/2 MIC. (**A**) Colony morphology of *M. furfur* CBS 1878 on YPD-Tween and IM agar. The plates were incubated at 30 °C. Bars corresponding to 1 mm; (**B**) Growth kinetics of *M. furfur* CBS 1878 cultured in YPD-Tween broth and IM broth. Values represent the mean ± SD of OD at 600 nm (n = 3 in each group). Asterisks indicate statistical significance between groups (* *p* < 0.05); (**C**) Cell morphology of the yeast phase of *M. furfur* CBS 1878 cultured in IM broth (magnification × 630). Bars corresponding to 20 μm; (**D**) Cell morphology of the mycelial phase of *M. furfur* CBS 1878 grown in IM broth (magnification × 630). Bars represent 10 μm. Black arrows indicate multiple massive vacuoles. White arrows illustrate intense fluorescence of chitin-rich bud scars or hyphal tips.

Rhizomes from the *Zingiberaceae* species are particularly popular in Thaï folk remedies to treat *Malassezia*-related skin infections [121]. In 2021, Juntachai and Laokor investigated the antifungal potency of *Zingiberaceae* extracts. Among the tested panels, *A. galanta n*-hexane extract containing 83% of (2,6-dimethylphenyl)borate was the most active fraction with MIC ranging from 0.04–0.08 mg/mL against a panel of 6 different *M. furfur* strains. In addition to the low content of hexadecenoic acid (4%) and 1,8-cineol (2.6%), other lower content components were also recorded (Figure 22). Due to the reported activity of long chain fatty acids, hexadecanoic acid could play a role in the observed antifungal activity. This is even at low concentrations. Its mode of action was elucidated using microscopic and macroscopic cell observation. It relies on an inhibitory effect on virulent hyphal growth (Figure 23).

### 3.8. Active Compounds without Known Biological Targets

Compounds, active against *Malassezia* species but without indication of mode of action, will be discussed in this subpart. Natural compounds, biosourced derivatives, and synthetic compounds are divided into three categories.

#### 3.8.1. Synthetic Anti-*Malassezia* Compounds

**Figure 24:**
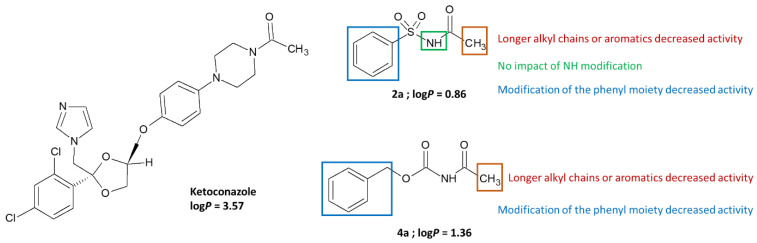
Structures of HIT compounds **2a** and **4a** and ketoconazole with some structure–activity relationships and logP (adapted from [122]).

A novel protocol for the *N*-acylation of sulfonamides and carbamates with carboxylic acid anhydrides under solvent-free conditions was reported by Kumar Thulam et al. [122]. The synthesized *N*-acyl drug candidates were successfully evaluated as antifungal agents against *M. furfur* and *M. pachydermatis* using ketoconazole as a reference. Compounds **2a**, **4a** exhibited MIC = 10 µM, being more active than ketoconazole (MIC around 95 µM for both strains) (Figure 24). The lipophilicity of compounds **2a** and **4a**, expressed by their logP, is lower than ketoconazole. It is notable that modification of the NH did not impact biological activity, whereas chemical modifications of the aryl or alkyl moieties dramatically decreased antifungal potency [122]. Structure–activity relationships are summarized in Figure 24. No mode of action for such compounds has been hypothesized but these compounds are structurally similar to β-carbonic anhydrase inhibitors.

**Figure 25:**
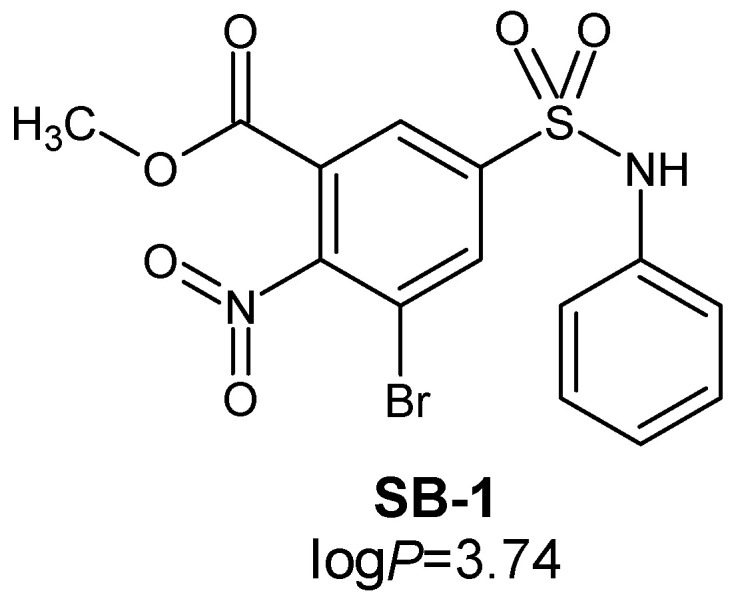
Chemical structures of SB-1.

In a similar manner, Gruzman, Cohen et al. described the 8-step synthesis of novel sulfamoylbenzoates and their subsequent inhibition of *M. furfur* growth. Methyl-3-bromo-2-nitro-5-(*N*-phenylsylfamoyl)benzoate SB-1 exhibited significant cytotoxic activity against *M. furfur* and was chosen as HIT for further development in the area of seborrheic dermatitis drugs (Figure 25) [123].

#### 3.8.2. Bio-Inspired Compounds

Green silver nanoparticles (AgNPs) were synthesized using *Coriandrum sativum* leaf extract. Flavonoids present in the extract serve as reducing agents for nanoparticle formation. A particle size of 37 nanometers was measured. The AgNPs antifungal activity was measured against *Malassezia furfur* MTCC 1374 and displayed a MIC = 25 µg/mL [124]. Rao and Paria [125] also reported the antifungal activity of *Aeglemarmelos* leaf extract mediated AgNPs against *M. furfur.* They reported a 20.5 mm inhibition zone at 6.8 μg/mL concentration. A review on the use of natural-product-based nanomedicine has recently been published, which helps to rationalize such development [126].

#### 3.8.3. Natural Compounds

Numerous reports and some reviews deal with natural compounds used as antifungals against *Malassezia* species without describing their mode of action [127,128]. The mode of action of natural compounds has already been hypothesized based on other yeasts, such as *Candida* spp. These include the permeabilization of the membrane, DNA, and mitochondrial damage [129]. In this subpart, selected examples will be detailed to show the richness of chemical structures from bioresources. 

**Figure 26:**
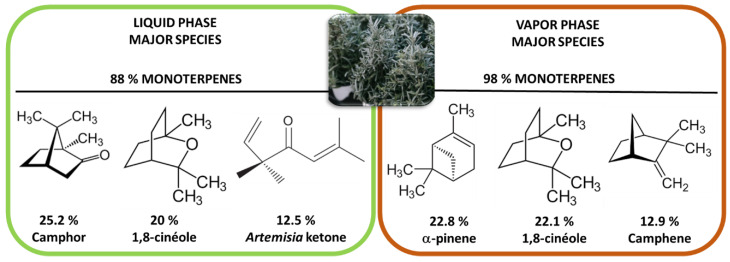
Major components of liquid and vapor phases of *Artemisia annua* essential oil.

An investigation by Santomauro et al. reported that *Artemisia annua* essential oil had antifungal activity against a panel of *Malassezia* species, both in liquid and vapor phases [130]. Characterization of the essential oil revealed monoterpenes as the major compounds in both phases (Figure 26). Essential oil of *A. annua*, both in liquid and vapor phase, exhibited significant antimicrobial activity towards almost all the twenty strains of *Malassezia* tested. For most strains, the MIC ranged from 0.78 µL/mL to 1.56 µL/mL. Moreover, the MIC for the vapor diffusion assay was lower than those obtained by the liquid method.

**Figure 27:**
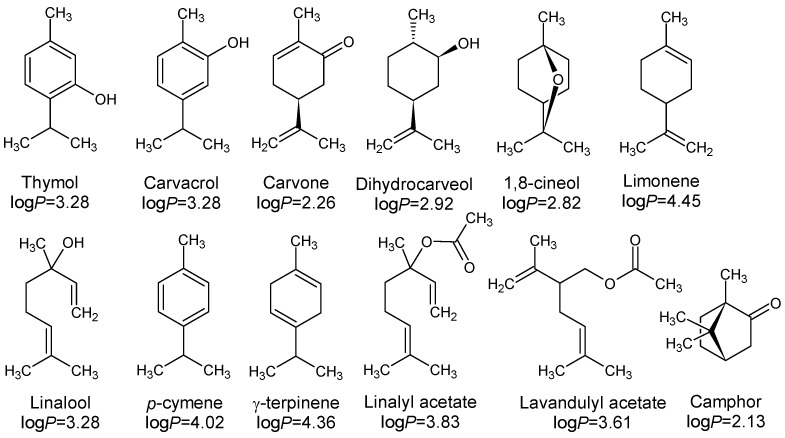
Chemical structures of components of essential oils studied by [131].

The essential oils of four aromatic plants, e.g., *Origanum vulgare* subsp. hirtum, *Mentha spicata*, *Lavandula angustifolia*, and *Salvia fruticose*, exhibited antifungal properties against *M. furfur* without any mutagenic activity detected by the Ames test [131]. The chemical composition of each essential oil was determined. Table 4 summarizes the main components of each compound, along with the diameter of inhibition measured by disk diffusion against *M. furfur*. Among the 4 essential oils tested, *Origanum vulgare* subsp. *Hirtum* was the most effective growth inhibitor against *M. furfur* with a zone of inhibition of 21 mm. Based on its composition, thymol and carvacrol were identified as its main active ingredients. Interestingly, carvacrol and thymol share strong chemical properties (Figure 27), which means they are both natural phenols.

Celery essential oil at 1% and its volatile vapor diluted at 1 mL/800 mL air inhibited the *M. furfur* growth [132]. However, the exact composition of such celery essential oil was not determined. *Usnea* sp. methanol extract (1 mg/disc) was reported by Rukayadi et al. as fair antifungal against *M. furfur* ATCC 14521 [117]. These fruticose thalli lichen inhibited growth of *M. furfur* with inhibition zone diameter of 34 mm and MIC and minimum fungicidal concentration (MFC) values of 16 and 64 μg/mL, respectively [133,134]. Notably, all identified chemical components displayed high lipophilicity, as expressed by their logP. 

Maboni et al. as reported that propolis extract was active against *M. pachydermatis* with a MBC_90_ = 5.3 mg/mL [135]. In this study, the ethanol and butanol fractions of white rose petal extract were evaluated for their antifungal activities against *M. furfur*. They exhibited strong MICs of 1.5 mg/mL and 0.5 mg/mL for WRPE-EtOH and WRPE-BuOH, respectively [136].

Naeini et al. reported in 2011 the evaluation of essential oils (EO) derived from water-distillation from three Iranian medicinal plants against 29 different *Malassezia* strains. The chemical composition of *Zataria multiflora*, *Pelargonium graveolens* and *Cuminum cyminum* EOs was fully characterized and revealed that the main oil components were carvacrol (61.3%) and thymol (25.2%) for *Z. multiflora*, α-pinene (30%), limonene (21%) and 1,8-cifor *C. cyminum* and citronellol (28.2%) and geraniol (22.1%) for *P. graveolens* (Table 5). The activity was measured by the disk diffusion method and expressed as the zone of inhibition (ZOI in mm). In comparison with ketoconazole as a reference, *Malassezia* spp. showed a high susceptibility to the three extracts [137]. No information was provided about the mode of action of these EOs.

A review by Bilia et al. detailed the biological activity of a variety of plant essential oils against a panel of *Malassezia* species [138]. Table 6 summarizes the main chemical components identified in the essential oil towards their measured activity against *M. furfur*.

Table 7 presents the same data but extended to seven different *Malassezia* strains. As described before, all active compounds exhibited high liphophilicity as expressed by logP.

All these data highlight the potential clinical applications of essential oils and natural resources against Malassezia species. In essential oils, active compounds come from different chemical families, such as terpenes and phenols, but they share some similarities, in particular their high lipophilicity. Indeed, the logP of each ingredient ranges from 1.90 for thujone to 6.78 for β-caryophyllene. For the highly lipophilic Malassezia yeast, this characteristic is essential.

To go further, in 2020, Rajput and Kumar reviewed medicinal plants as a source of bioactive ingredients against pathogens targeting hair and scalp [128]. Kowalski also reported earlier the antimicrobial activity against *M. pachydermatis* of essential oils and extracts of the roseweed plants used by American Indians. These oils had an inhibitory effect comparable to Amphotericin B [145]. Other bioresources have been reported such as: (a) roots of *Asparagus racemosus Willd* [146], (b) antimicrobial antibiotics from soil *Streptomyces* sp. SCA 7 [147], (c) leaf and rhizome of *Hedychium coronarium*, active thanks to its antioxidative capacity [148], (d) seed and leaf from *Alpinia speciosa* cultivated in Taiwan [149], (e) *Ilex paraguariensis* [150], (f) apricot seed [151], and (g) *Vitis vinifera* seeds [152]. 

In summary, nature is a great inspiration for the discovery and development of new antifungals to treat *Malassezia* species.

## 4. Critical Review, Conclusions, and Perspectives

**Figure 28:**
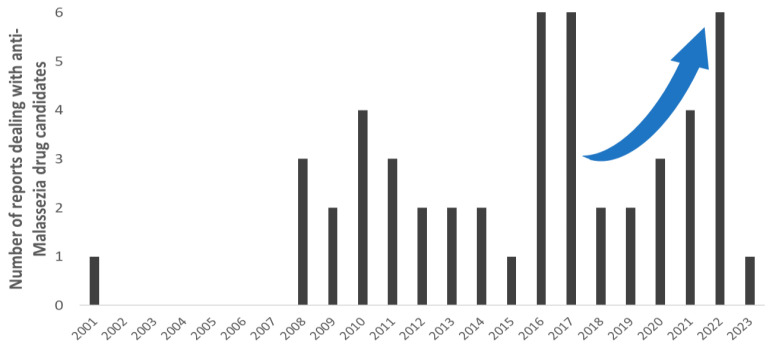
Timeline of reports dealing with anti-Malassezia drug candidates from 2001 to nowadays.

This review studies and compiles around fifty different reports. Over the past two decades, little research has been conducted on new compounds to tackle *Malassezia* spp. with a maximal occurrence of six papers per year (Figure 28). A growing number of papers is observed from 2018, possibly due to the emergence of resistant *Malassezia* strains, but this tendency needs to be confirmed.

Among them, 37%, i.e., 18 reports, presented active compounds without any clue about their mode of action against *Malassezia* spp. 

In terms of the source of active compounds, it is noteworthy that 63% of the drug candidates are bioresources such as essential oils or antimicrobial peptides. Over half of such research reports mention extracts from medicinal plants. Generally, the chemical composition of the extracts has been elucidated and the main components are known. 

**Figure 29:**
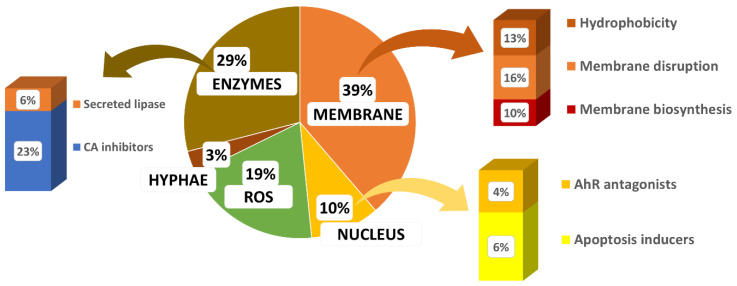
Repartition of the reported mode of action for active drugs.

From this review, we can draw the following conclusions:

1. Lipophilicity is the key. Indeed, natural active molecules are lipophilic, with logP ranging from 1.90 to 11.90, with a medium value around 3–4. Moreover, synthetic organic compounds also displayed high lipophilicity. This criterion can be adjusted by formulation if the drug is incorporated into lipidic nanoparticles, which favors skin penetration and decreases side-effects.
2. Chemically speaking, two classes of molecules could be developed. These are (i) heteroatomic ones, including selenium or boron, with an adjusted HLB; and (ii) amphiphilic compounds with a high ability to disrupt the thick cell membrane.
3. Biological targets remain underexploited. Based on the reported mode of action, it appears (Figure 29) that β-carbonic anhydrase inhibitors and antioxidant compounds are the most common. Drug candidates mainly target membranes. Three targets are under-valued in anti-Malassezia drug development, AhR antagonists, inhibitors of secreted lipase, and biofilm inhibitors.

4. Nature is a source of inspiration for the development of bio-inspired optimized drugs to selectively target *Malassezia* spp.

Overall, the growth of resistant strains of *Malassezia* is of current interest, which calls for the development of new strategies, relying on the virulence factors of this organism, to prevent an uncontrollable spread of human pathogenic strains. Among the various strategies that could be employed, the development of AhR antagonists, the inhibition of the secreted lipase, as well as the fight against the development of biofilms sound particularly interesting. The hydrophily/lipophily balance of a drug candidate should also be considered within this context, as well as within lipophilic yeast being the dominant cause of skin infections within a skin disease context, in order for the drug candidate to be successful in skin penetration and killing lipophilic yeast. 

## Figures and Tables

**Table 1:** *Malassezia* species isolated from human skin.

*Malassezia* Species	References
*M. restricta*	[13]
*M. globosa*	[14]
*M. furfur*	[15]
*M. dermatis*	[16]
*M. arunalokei*	[17]
*M. slooffiae*	[14]
*M. sympodialis*	[18]
*M. obtusa*	[13]
*M. yamatoensis*	[19]
*M. japonica*	[20]

**Table 2:** Targeting cell wall biosynthesis.

Biological Target	Active Compounds
Fungal ergosterol synthesis inhibitors	Azoles (ketoconazole, itraconazole, fluconazole, voriconazole, posaconazole…)
Squalene epoxidase inhibitors	Morpholines: amorolfine
	Allyamines: terbinafine, butenafine
Ergosterol disruptors (membrane permeabilization)	Polyenes antibiotics: amphotericin B or nystatin
Glucan synthesis Inhibitors	Echinocandins, caspofungine, micafungine, anidulafungin
Chitin synthesis inhibitors	Nikkomycin, Polyoxins

**Table 3:** MIC_50_ (mg/mL) and MFCs (mg/mL) of fermented *O. sanctum* extract (FE) against four strains of *M. furfur*.

Samples	*M. furfur* 133		*M. furfur* 656		*M. furfur* 6000		*M. furfur* 7966	
	MIC_50_	MFCs	MIC_50_	MFCs	MIC_50_	MFCs	MIC_50_	MFCs
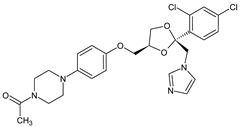 Ketoconazole	0.0078	0.0078	0.25	0.25	0.0625	0.0625	0.125	0.125
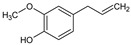 Eugenol (main component of FE)	0.125	0.125	0.25	0.25	0.125	0.5	0.125	0.5

**Table 4:** Composition of extracts and activity of the pure compound against *M. furfur*.

Entry		ZOI * (mm)	* Origanum vulgare * subsp. *hirtum*	* Mentha * * spicata *	* Lavandula * * angustifolia *	* Salvia * * fruticose *
1	Thymol	19	45.22	-	-	0.03
2	Carvacrol	15	33.05	-	-	-
3	Carvone	7	-	59.12	-	-
4	dihydrocarveol	9	-	6.87	-	-
5	1,8-cineole	2	0.21	5.42	13.10	43.10
6	Camphor	2	-	-	5.50	18.34
7	linalool	0	0.18	0.06	20.18	0.12
8	p-cymene	1	7.35	-	0.09	0.93
9	γ-terpinene	1	5.54	0.04	0.07	0.09
10	Linalyl acetate	0	-	-	18.60	0.07
11	Lavandulyl acetate	ND **	-	-	16.01	-
12	limonene	0	1.21	5.02	0.25	1.50
13	Essential oil ZOI (mm)		21	0	3.5	0

* Zone of inhibition measured in millimeters in the disk diffusion assay. ** ND is used for Not Determined.

**Table 5:** Chemical structures and activities of essential oils from [137].

	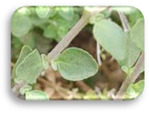 *Zataria multiflora*	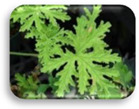 *Pelargonium graveolens*	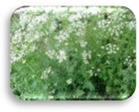 *Cuminum cyminum*
	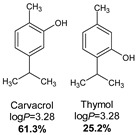	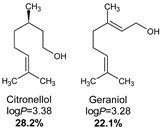	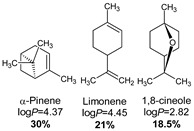
Mean ZOI (mm)	28.1	26.1	48.3
Lower ZOI (mm)	20	10	30
Higher ZOI (mm)	>50	>50	>50

**Table 6:** Main components and biological activity against *M. furfur* for a panel of essential oils.

Resource		Main Chemical Components	MIC	Reference
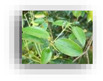	*Cinnamomun zeylanicum* Blume	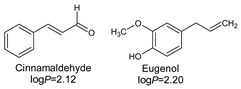	32 µg/mL	[139]
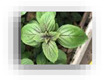	*Ocimum kilimandscharicum* Gürke	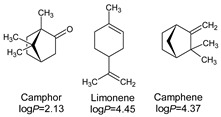	128 µg/mL	
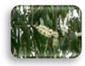	*Malaleuca leucadendrun* (L.) L.	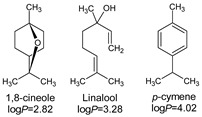	64 µg/mL	
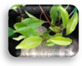	*Syzygium aromaticum* (L.)	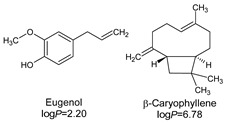	0.625 µL/mL	[140]
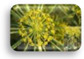	*Thapsia villosa* (L.)	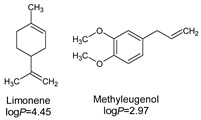	2.5 µL/mL	[141]
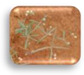	*Deverra tortuosa* subsp. *arabica* Chrtek, flowers	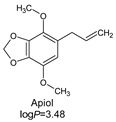	5.0 µL/mL	[142]
	*Deverra tortuosa* subsp. *arabica* Chrtek, stem		8.0 µL/mL	
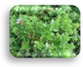	*Origanum vulgare* L.	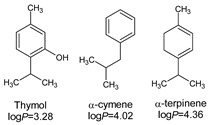	780 µg/mL	[143]
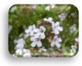	*Thymus vulgaris* L.	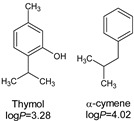	920 µg/mL	

**Table 7:** Main components and biological activity against different *Malassezia* strains for a panel of essential oils. * is for µL/mL.

			*M. furfur*	*M. sympodialis*	*M. slooffiae*	*M. globosa*	*M.obtusa*	*M. nana*	*M. restricta*	
Bioresource		Main Chemical Components	MIC (µg/mL or *µL/mL)							Reference
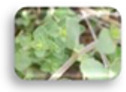	*Zataria* *multiflora* Boiss.	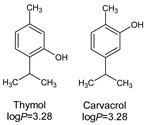	35	30	80	50	60	30	40	[127]
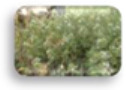	*Thymus* *kotschyanus* Boiss.		60	60	80	80	80	30	110	
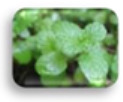	*Mentha spicata* L.	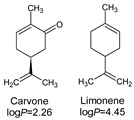	125	100	100	250	85	65	85	
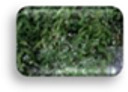	*Artemisia sieberi* Besser	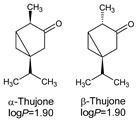	250	85	150	50	155	110	100	
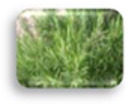	*Salvia* *rosmarinus* Schleid.	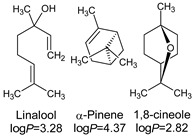	260	250	420	410	850	100	350	
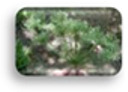	*Heracleum persicum Desf.* ex Fisch.	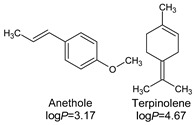	125	250	480	250	560	75	320	
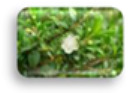	*Myrtus communis* L.	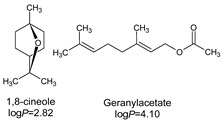	31 *	62	31	31	62	-	125	[144]

## Data Availability

No new data have been created. This is a critical review paper.
